# Intensive End-of-Life Care: Implementation of a Canadian Guideline-Based Order Set for the Withdrawal of Life-Sustaining Therapy in the Intensive Care Unit

**DOI:** 10.1089/pmr.2024.0091

**Published:** 2025-04-10

**Authors:** Alison Knapp, Jennifer M. O’Brien, Maria Cruz, Mary Ellen Walker, Joann Kawchuk, Carol Brons, Sabira Valiani

**Affiliations:** ^1^ Provincial Department of Anesthesiology, College of Medicine, University of Saskatchewan, Saskatoon, Canada.; ^2^ Patient and Family Partner, Saskatchewan Center for Patient Oriented Research, Saskatoon, Canada.; ^3^ Department of Medicine, College of Medicine, University of Saskatchewan, Saskatoon, Canada.

**Keywords:** critical care, end-of-life care, ICU, implementation science, quality improvement, withdrawal of life-sustaining therapy

## Abstract

**Background:**

An increasing number of patients receive end-of-life care in the intensive care unit (ICU). Death often occurs in the ICU after a decision has been made to withdraw life-sustaining therapies. In 2016, Downar et al. published Canadian consensus guidelines to standardize practices for withdrawal of life-sustaining therapy in the ICU. In this study, we sought to understand the feasibility and acceptability of implementing an order set, nursing flowsheet, and nursing care plan based on these guidelines in two ICUs in Saskatchewan, Canada.

**Methods:**

We used a hybrid effectiveness-implementation design, engaging a steering committee of ICU health care providers and leadership to guide implementation. We conducted a six-month pilot implementation. We collected data in the three months pre-implementation, during the six-month implementation period, and for three months post-implementation. To evaluate implementation outcomes, we used the Consolidated Framework for Implementation Research to develop semi-structured interviews and feasibility surveys. To measure effectiveness outcomes, bedside nurses completed Quality of Death and Dying surveys, and we performed a patient chart review.

**Results:**

The intervention materials added to the burden of paperwork of bedside health care providers but helped them provide quality end-of-life care, meet the needs of patients and their families, and lessen ethical tensions between symptom control and hastening death. There was no difference in cumulative sedative dosing and time to death after extubation in the pre-implementation, implementation, or post-implementation periods. A significant increase in symptom assessment (pain, dyspnea, and agitation) using standardized tools was observed during the implementation and post-implementation periods. There was an improvement in holistic care outcomes post-implementation.

**Conclusions:**

We implemented current Canadian best-practice guidelines for providing end-of-life care in the ICU using a multidisciplinary approach. This study offers insight into how standardized symptom assessment and medication titration can be incorporated into the complex ICU environment.

## Introduction

In the intensive care unit (ICU), death is most likely to occur after a decision has been made to withdraw life-sustaining therapy (WLST).^1,2^ Quality end-of-life care during WLST poses a unique set of challenges.^3,4^ First, the withdrawal of mechanical ventilation can result in distress to patients due to dyspnea and difficulty with secretion clearance.^1,4^ Second, perceived ethical tensions can occur when the dying patient has the potential to donate organs after cardiac death is declared.^5^ Finally, within the technologically intense ICU environment, making space for end-of-life care that honors patients’ and their families’ identities and values is important.^3,6^ These challenges highlight the need for transparent, consistent, and compassionate provision of symptom management and supportive holistic care at the end-of-life in the ICU.^3,6,7^

In 2016, a Canadian consensus guideline for WLST in the ICU was published.^7^ This guideline differed from practice in our local center by recommending the use of (1) symptom assessments using validated pain, dyspnea, and sedation scales; (2) specific titration guidelines for opiate and sedative medications; and (3) stepwise withdrawal of mechanical ventilation to ensure patient comfort. Providing high-quality end-of-life care in line with these guidelines involves standardized assessment and treatment of symptoms for dying patients, which requires a commitment from all members of the multidisciplinary health care team. The implementation of Canadian consensus guidelines for WLST in the ICU has not been studied.

## Objective and Hypothesis

We sought to evaluate the implementation of the Canadian consensus guidelines for WLST in two ICUs in Saskatchewan, Canada. We hypothesized that implementation of guideline-based care would be feasible and safe.

## Methods

### Design

**FIG. 1.**
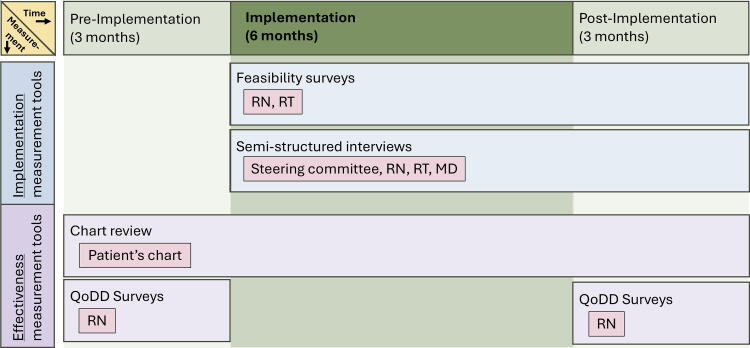
Study design. The figure illustrates the study timeline (top, green bar) and the measurement tools utilized during each time period (blue and purple bars). Data sources for each measurement tool are indicated in pink boxes. RN, registered nurse; RT, respiratory therapist; MD, medical doctor.

This study followed a pre–post, hybrid effectiveness-implementation design (Fig. 1).^8,9^ Effectiveness-implementation designs allow for simultaneous evaluation of an implementation strategy (e.g., engagement of a multidisciplinary team, educational initiatives) and relevant clinical outcomes (e.g., symptom control).^9^ We evaluated implementation with a feasibility survey and semi-structured interviews, based on relevant domains and constructs from the Consolidated Framework for Implementation Research (CFIR, Supplementary Data S1).^10^ We evaluated effectiveness outcomes by collecting the results of Quality of Death and Dying (QoDD) surveys from bedside nursing staff and by completing a chart review.^11^

### Steering committee

To oversee implementation of the Canadian WLST guidelines, we created a Steering Committee. The Steering Committee consisted of two patient family partners, ICU leadership, and multidisciplinary ICU team members (e.g., nurse managers, nurse educators, respiratory therapist [RT] educators, physician site leads, ICU pharmacists, and social workers). This Steering Committee acted as a research partner by providing feedback on the content and method of implementation and disseminating culture change within the ICU.

### Intervention overview

#### Intervention development

One physician (S.V.) compared the pre-implementation ICU order set and local practice for WLST to Canadian guidelines.^7^ Guidelines differed from pre-implementation WLST practices by suggesting or recommending (1) routine nursing assessments of agitation, pain, and dyspnea using validated scoring systems;^12–15^ (2) an opioid and sedative titration protocol based on symptom assessments; and (3) a ventilator weaning protocol. Local pre-implementation practice instead relied on nursing expertise and subjective symptom assessment for dosing and titration of infusions and boluses of opioids and sedatives.

Next, health care providers (HCPs) on the research team collaboratively developed intervention materials with the Steering Committee to incorporate these guidelines into local practice. The final intervention materials comprised (1) an order set, (2) a nursing flowsheet, and (3) a nursing care plan. We chose symptom assessment tools based on HCPs’ familiarity with the tool and ease of use. The Critical Care Pain Observation Tool (CCPOT) and Richmond Agitation Sedation Score (RASS) are validated for the ICU setting, recommended by Canadian guidelines, and were used locally to assess pain and sedation in nonpalliative ICU patients.^12,13^ Initially, the Respiratory Distress Observation Scale was chosen based on Canadian guidelines, but after further discussion with the respiratory therapy and nurse educators, a simplified version of this scale was ultimately implemented (modRDOS-4).^14^ Opioid and sedative infusion and bolus titration were drafted based on Canadian guidelines and integrated into the order set with the assistance of an ICU pharmacist. Finally, guidelines suggest using a ventilator weaning protocol but were not prescriptive. Therefore, we developed a local protocol based on a literature review and collaborative discussions with the respiratory therapy educator.^16–18^

Two drafts of intervention materials were created and circulated broadly to bedside ICU healthcare providers (HCPs), palliative care physicians, donation physicians, ICU leadership, and the Steering Committee before intervention materials were finalized and implemented. The preexisting ICU order set and developed intervention materials are included in Supplementary Data S4 and S5, respectively.

#### Implementation

Prior to implementation, members of the Steering Committee and the senior author provided presentations and bedside education to the multidisciplinary ICU team. Educational initiatives introduced the importance of the project and provided an orientation to the intervention materials. Sustained support was available through Steering Committee members (particularly, nursing educators) during and after the implementation period. Implementation commenced in May 2022 for a planned six-month period.

### Data collection

Data were collected during the three months prior to implementation (pre-implementation period), during the six-month implementation period (implementation), and for three months after the implementation was completed (post-implementation period).

We conducted semi-structured interviews and feasibility surveys to assess CFIR domains identified *a priori* as important potential barriers and facilitators to implementation (see *Design*, above). The semi-structured interview guide (Supplementary Data S2) and feasibility survey (Supplementary Data S3) were created by adapting standardized questions from the CFIR tools and templates available online (cfirguide.org). A research assistant (M.C.) conducted interviews with bedside HCPs and Steering Committee members by phone or video conference, which lasted approximately 40 minutes. Feasibility surveys were distributed to all bedside nurses whose patients underwent WLST and by email to RTs.

Effectiveness outcomes were collected using the QoDD surveys through patient chart reviews. The QoDD survey was originally developed using a conceptual framework of death and dying and validated using interviews with patient family members.^19,20^ Subsequently, the survey was adapted to measure nurses’ perception of symptom control and holistic care for patients during WLST in the ICU.^21,22^ We undertook chart reviews to assess safety and compliance with charting of symptom assessment and treatment. We screened charts of all patients who died in the specified time period(s). Patients were included if they died in the ICU during the specified time frames. Patients were excluded if a WLST order set had not been completed, and the reasons why this order set was not completed were also documented (including death after a cardiac arrest/code blue, brain death, medical assistance in dying, or reason unclear). Safety was measured by determining the cumulative doses of narcotics and sedatives administered and time to death from ventilator withdrawal for intubated patients.

All participants provided informed consent prior to completing surveys or semi-structured interviews. HCPs received a $5 coffee voucher for completing surveys (QoDD and feasibility) and a $20 coffee voucher for participating in a semi-structured interview.

### Data analysis

Semi-structured interviews were audio recorded and transcribed verbatim using Zoom. Participants were invited to review their interview transcripts and make changes or additions. Transcripts were coded deductively by at least two team members according to CFIR framework constructs. We then combined all quotes related to each CFIR construct and inductively coded these quotes into themes. Themes were categorized as barriers and facilitators at two collaborative meetings. Trustworthiness and rigor were ensured through an iterative analysis, constant comparison of emerging themes against raw transcripts, and engagement with the Steering Committee to ensure results were reflective of their experience.

Feasibility survey responses were collated and reported as percentages. The responses were aggregated and organized into their associated CFIR domain. We compared qualitative comments from feasibility surveys to the results of the semi-structured interviews and added or expanded themes where necessary.

QoDD surveys were analyzed with *p*-values generated from Mann–Whitney U tests for continuous variables and likelihood ratio tests for categorical variables (Table 1). Questions 1 through 10, 11b, 12b, 13b, and 14b involved a Likert scale rating patient experience from 0 (terrible experience) to 10 (almost perfect experience), and median values with interquartile ranges were reported. Questions 11a, 12a, 13a, and 14a were reported as frequencies.

**Table 1.** Quality of Death and Dying Survey Results Pre-Implementation and Post-Implementation.

Question	Pre-implementation (*n* = 74) median (IQR)/*N* (%)	Post-implementation (*n* = 34) median (IQR)/*N* (%)	*p* value
1. Having control of his/her pain	9.0 (7.0 to 10.0)	9.0 (8.0 to 10.0)	*p* > 0.05
2. Having control over what was going on around him/her	5.0 (3.0 to 9.0)	7.0 (3.5 to 9.0)	*p* > 0.05
3. Breathing comfortably	8.0 (6.0 to 9.0)	8.0 (7.0 to 9.8)	*p* > 0.05
4. Keeping his/her dignity and self-respect	9.0 (7.0 to 10.0)	9.0 (8.0 to 10.0)	*p* > 0.05
5. Spending time with his/her spouse or partner	8.0 (4.3 to 10.0)	9.0 (7.8 to 10.0)	^*^p < 0.05
6. Spending time with his/her children	8.0 (2.3 to 10.0)	9.0 (7.0 to 10.0)	^*^p < 0.05
7. Spending time with other family and friends	8.0 (4.5 to 9.3)	9.0 (7.8 to 10.0)	^*^p < 0.05
8. Being touches or hugged by loves ones	8.0 (5.0 to 9.0)	9.0 (7.0 to 10.0)	^*^p < 0.05
9. Having one or more visits from a religious or spiritual advisor	8.5 (5.3 to 9.0)	9.5 (9.0 to 10.0)	^*^p < 0.05
10. Having a spiritual service or ceremony before his/her death	8.0 (5.0 to 10.0)	10.0 (9.0 to 10.0)	^*^p < 0.05
11a. Was anyone, including family, friends or staff, present at the moment of your patient’s death?	Yes—67 (90.5%)	Yes—30 (88.2%)	*p* > 0.05
	No—2 (2.1%)	No—0 (0.0%)	
	Don’t know 3 (3.2%)	Don’t know—2 (5.9%)	
	Missing—2 (2.1%)	Missing—2 (5.9%)	
11b. How would you rate this aspect of your patient’s death?	9.0 (7.3 to 10.0)	9.0 (8.0 to 10.0)	*p* > 0.05
12a. In the moment before your patient’s death, was s/he:	Awake—0 (0.0%)	Awake—0 (0.0%)	*p* > 0.05
	Asleep—7 (9.5%)	Asleep—4 (11.8%)	
	Coma—63 (85.1%)	Coma—28 (82.4%)	
	Missing—4 (5.4)	Missing—2 (5.9%)	
12b. How would you rate this aspect of your patient’s death?	9.0 (8.0 to 10.0)	9.0 (8.5 to 10.0)	*p* > 0.05
13 a. Did your patient receive mechanical ventilation during his/her stay in the ICU?	Yes—67 (90.5%)	Yes—29 (85.3%)	*p* > 0.05
	No—5 (6.8%)	No—5 (14.7%)	
	Missing—2 (2.7%)		
13b. How would you rate this aspect of your patient’s dying experience?	8.0 (6.0 to 10.0)	8.5 (7.0 to 10.0)	*p* > 0.05
14a. Do you think that your patient received the right amount of sedation during his/her stay in the ICU?	Yes—61 (82.4%)	Yes—28 (82.4%)	*p* > 0.05
	No—11 (14.9%)	No—5 (14.7%)	
	Missing—2 (2.7)	Missing—1 (2.9%)	
14b. How would you rate this aspect of your patient’s dying experience?	9.0 (8.0 to 10.0)	9.0 (8.0 to 10.0)	*p* > 0.05^*^

ICU, intensive care unit; IQR, interquartile range.

^*^ *p* < 0.05, statistically significant.

For the chart review, data were summarized with medians, interquartile ranges, counts, and percentages due to its nonparametric distributions. Friedman analysis of variance and Wilcoxon signed rank tests were used to assess differences in the rank distributions between the pre-, during, and post-intervention groups. Chi-squared tests were used to assess if the intervention group category had significant associations with other categorical variables. Likelihood ratio tests were used if assumptions for the chi-square test were not met. Statistical significance was evaluated at 0.05. Results were produced with IBM SPSS Statistics (Version 28).

## Results

### Implementation barriers and facilitators

#### Feasibility surveys

**FIG. 2.**
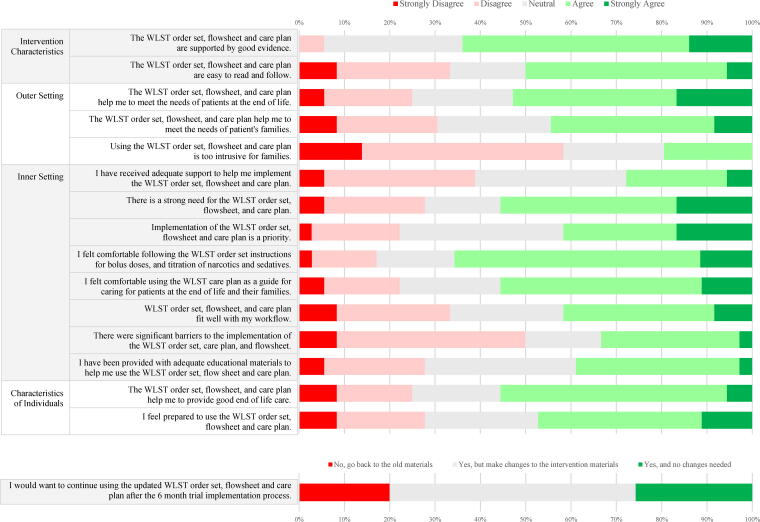
Nursing feasibility survey results grouped by Consolidated Framework for Implementation Research domain.

**FIG. 3.**
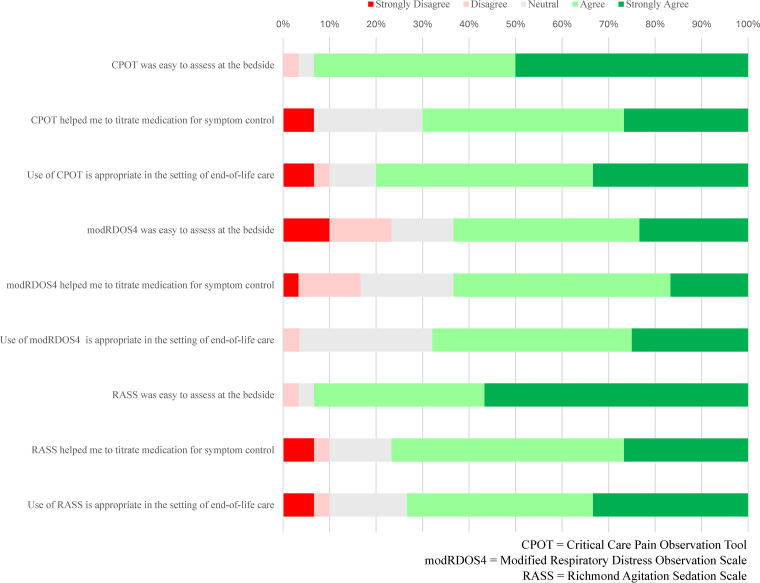
Nursing feasibility and acceptability of symptom assessment using standardized tools.

Forty feasibility surveys were completed by bedside nurses (*N* = 36) and RTs (*N* = 4) during the six-month implementation period. Complete results of the feasibility surveys for bedside nurses are included in Figures 2 and 3.

In caring for their patients, most bedside nurses reported that the intervention materials helped meet patients’ needs at the end-of-life and helped them provide good end-of-life care (52% and 56% agreed or strongly agreed, respectively). Bedside nurses generally agreed or strongly agreed (60% or more) that the symptom assessment tools (CCPOT, RASS, and modRDOS-4) were easy to assess, helped to titrate medications for symptom control, and were appropriate in the end-of-life setting (Fig. 3).

Bedside nurses identified gaps in the support of the implementation and integration into their workflow. Only 28% and 39% of bedside nurses strongly agreed or agreed that they had been provided with adequate support and educational materials, respectively. Furthermore, only 42% of nurses agreed or strongly agreed that the intervention materials fit well with their workflow.

Regarding the quality of the intervention materials themselves, bedside nurses generally agreed or strongly agreed that the intervention materials were supported by good evidence, were easy to read and follow, and felt comfortable using the intervention materials for titration of symptom control medications (64%, 50%, and 66%, respectively). Most bedside nurses (54%) wished to continue with the intervention materials after the pilot implementation, but with some changes. Only 20% of bedside nurses wanted to revert to the prior order set, and 26% were happy to continue with the intervention materials without changes.

Due to low response rates (*n* = 4), drawing conclusions from the RT feasibility survey is difficult. However, most responses to feasibility statements were in the neutral to positive range. The feasibility survey was similar to that administered to the bedside nurses and assessed the respondent’s agreement with whether the implementation of the intervention helped provide good end-of-life care, was well supported, and was easy to use.

#### Semi-structured interviews

Ten semi-structured interviews were conducted with members of the Steering Committee (*N* = 4) and bedside HCPs (physicians, nurses, and RTs) (*N* = 6).

##### Facilitators

1. *Caring for the patient.* Bedside HCPs and steering committee members believed the intervention was important and aligned with principles of patient and family-centered care. For bedside HCPs, embedded symptom assessment tools for pain, agitation, and dyspnea were easy to use and integrated well into bedside care. Bedside HCPs found benefit in objective, standardized assessment and treatment of symptoms. Standardized symptom assessment tools also helped with communication with family members. A bedside HCP highlighted how the order set was particularly helpful with communication with family: *“… a light bulb turned on. It was kind of nice that I had these terms that I could ask…it helps me guide my questions to family when I’m trying to dose medications.”* (Participant 5).
2. *Ethical tensions.* Bedside HCPs and steering committee members described a relief in tension between providing symptom management and hastening death. One Steering Committee Member described how, prior to implementation of the intervention, nurses sometimes felt uncomfortable managing symptoms due to the proximity between medication administration and death: *“The fact that before you didn’t have a measurable thing to give narcotics to or sedation to that was in conflict with people. Because they felt that they were causing death instead of just managing symptoms.”* (Participant 4).
3. *Health care provider characteristics and team dynamics.* Bedside HCPs welcomed continuing professional development and described themselves as adaptable to change. Participants commented that the intervention materials were particularly helpful for HCPs less familiar with WLST in the ICU. Bedside HCPs reported that the Steering Committee was a valuable champion of the implementation and effectively provided the necessary educational support and communication.

##### Barriers

1. *Caring for the patient*. Bedside HCPs spoke of the tension between providing individualized care to meet the needs of patients and their families and the standardized protocols introduced by the order set. They expressed the importance of clinical experience in individualizing care at the end-of-life and meeting the family and patient’s unique needs.
2. *Workflow and resources.* Within the ICU environment, bedside HCPs described the length of the intervention materials and the limited resource of time as barriers to implementation. The intervention materials required nurses to document medication administration in both the medication administration record and the flowsheet, which was a barrier to efficiency and had the potential to cause confusion about the total dose of medication administered. Some bedside HCPs would have preferred an in-person debrief following the trial period.
3. *Values, beliefs, and characteristics of health care providers.* Some interviewed members of the Steering Committee and bedside HCPs felt the care during WLST was already excellent pre-implementation. Therefore, the need for a new process was less clear. Increased years of experience of the HCP was noted to be a barrier as it resulted in resistance to change.

### Effectiveness outcomes

#### Quality of death and dying surveys

A total of 108 QoDD surveys were collected. The results (*N* = 108) were analyzed pre-implementation (*N* = 74) and post-implementation (*N* = 34) and are presented in Table 1. There were no differences among the pre-implementation, implementation, or post-implementation groups in the perceived quality of pain control or comfort of breathing pre-implementation. There was significant perceived improvement in several holistic care outcomes, including spending time with a spouse or partner, spending time with children, spending time with other family and friends, being touched or hugged by loved ones, having a visit from a religious or spiritual advisor, and having a spiritual service or ceremony before death.

#### Chart review

We screened a total of 160 patient charts for eligibility in the pre-implementation, implementation, and post-implementation periods. Forty-four patient charts were included in the pre-implementation phase, 34 patient charts were included in the implementation phase, and 29 patient charts were included in the post-implementation phase. A WLST order set was not completed for unclear reasons in 25%, 17%, and 32% of charts in the pre-implementation, implementation, and post-implementation phases, respectively. We collected limited demographic data; age and sex were not significantly different between groups.

The time from extubation to death was analyzed as an index for patient safety. There were no significant differences, with a median time of 12 minutes (4.8–24.3) pre-implementation, 11 minutes (4.0 to 34.0) during implementation, and 17 minutes (10.0–32.0) post-implementation (*p* value = 0.102). Cumulative sedative and opioid dosing were also analyzed with no significant differences between time periods.

To assess the adherence to and uptake of the new flowsheet and care plan, nursing compliance with charting of pain, dyspnea, and agitation assessments was analyzed. In the pre-implementation period, only 2.3% of patients (1/44) had one documented assessment of pain, agitation, or dyspnea using a validated scoring system, compared to 73.5% (25/34, *p* < 0.001) and 55.1% (16/29, *p* < 0.001) of patients in the implementation and post-implementation groups. Similarly, only 2.3% of patients (1/44) had two or more documented assessments of pain, agitation, or dyspnea, compared with 67.6% (23/34, *p* < 0.001) implementation and 48.2% (14/29, *p* < 0.001) post-implementation.

## Discussion

The current study demonstrates that implementation of the Canadian WLST guidelines was feasible. Nurses agreed that symptom assessment tools were easy to use and appropriate in the ICU end-of-life care setting. Nurses were comfortable using the newly introduced titration protocols. We were able to show a significant increase in the number of patients who had documented assessments of pain, agitation, or dyspnea from the pre-implementation to the implementation period (2.3% vs. 73.5%, respectively). However, this proportion was not well sustained in the post-implementation period (55.1%). Most nurses wished to continue the intervention following the pilot, but with changes to the intervention materials. We also identified that a significant proportion of patients dying in the ICU do not have a WLST order set completed for unclear reasons (17–32%).

### Barriers and facilitators

Reduction in symptom assessment over time, variable use of the order set, and desire for changes to the intervention materials, as described above, can be explored in the context of barriers to implementation. Nurses reported a disruption to workflow as a barrier, including an increase in charting and paperwork. Some of this increased charting may have been related to the use of symptom assessment tools, but semi-structured interviews and communication with the Steering Committee also indicated other workflow inefficiencies (e.g., length of the care plan, documentation burden of medication administration). Previous research has not highlighted increased workload as a potential barrier to the provision of quality end-of-life care in the ICU.^23,24^

Perhaps the most interesting facilitator of implementation was a reduction in the ethical tensions between providing symptom control and hastening death at the time of WLST in the ICU. Objective symptom assessment using validated tools and medication titration protocols increased nursing comfort in medication titration for symptom treatment. Previous research and guidelines have highlighted this ethical tension, especially in the setting of potential donation after cardiac death.^5,7,25^ Reducing ethical tension between symptom control and hastening death has the potential to reduce moral distress for bedside nurses.

Finally, individual HCP and team beliefs, values, and dynamics could be either facilitators or barriers. Semi-structured interview participants believed that the intervention materials would be most helpful to less experienced nurses and that more experienced nurses may be resistant to change. The multidisciplinary Steering Committee was engaged to champion the implementation process and facilitated communication, education, and support. Support for nursing staff, leadership, and good communication have been previously identified as facilitators to good end-of-life care.^23,26^ In the current reality of critical care nursing turnover, standardized order sets and strong leadership may be necessary to ensure continued excellence in end-of-life care in the ICU.

### Safety and effectiveness

Although not the primary outcome, we did capture limited safety and effectiveness outcomes using a chart review. We used cumulative sedative and opioid doses and time to death from extubation as a surrogate for safety.^27,28^ We demonstrated that cumulative doses of sedatives and opioids did not increase, and time to death from extubation did not change. QoDD scores for pain control, breathing comfort, and level of arousal also did not change following implementation. These findings, taken together, suggest that bedside nurses were administering medications for symptom control appropriately prior to implementation. Therefore, the strength of the implementation is in improving the process of transparent, objective assessment, and treatment of symptoms during the WLST period.

QoDD scores improved significantly for holistic end-of-life care outcomes in the post-implementation period. These outcomes included spending time with family and friends, more time being held or touched, and having a spiritual or service or ceremony before death. Improvement in holistic care was an unanticipated outcome of our study. It may relate to including prompts in the nursing care plan to discuss and offer such services to patients and families. Given the study design, these results should be considered hypothesis-generating only.

### Strengths

Strengths of our study include mixed methodology, use of the CFIR framework to study implementation systematically, and engagement of a multidisciplinary Steering Committee to act as a liaison between the research and clinical team. The combination of quantitative feasibility measurements and qualitative exploratory semi-structured interviews allowed us to understand the strengths and potential gaps in our implementation process. We utilized the CFIR framework to ensure that we captured qualitative and quantitative data related to all relevant domains of implementation.

### Limitations

There are a few limitations to this study. First, while our team recognized the importance of integrating family perspectives in the end-of-life care of ICU patients, we were unable to include the perspectives of the families of patients who died in the ICU during the implementation process due to resource constraints. We mitigated this limitation by including patient family partners on our research team and Steering Committee and intentionally including multidisciplinary HCPs with significant clinical experience within these teams.

Second, there is the potential for selection bias from the completed QoDD and feasibility surveys, as their completion was voluntary and may not reflect the experiences of all bedside HCPs. Finally, outcomes from the chart review and QoDD surveys are limited by the study design and cannot be considered conclusive.

## Conclusion

The current study demonstrates that implementation of Canadian best-practice guidelines for the provision of WLST in the ICU is feasible and safe. Despite certain limitations, this study offers insights into the importance of standardized, objective symptom assessment and treatment.

Standardized symptom assessment and treatment reduce ethical tensions between symptom treatment and hastening death and provide a mechanism for transparency in process. We suggest using a multidisciplinary team and minimizing disruptions to workflow.

## Ethical Approval and Consent

This study was approved by the Behavioural Research Ethics Board (Beh-REB no. 1518 approved December 19 2019).

## Abbreviations Used

CCPOT: Critical Care Pain Observation Tool
HCPs: health care providers
ICU: intensive care unit
RASS: Richmond Agitation Sedation Score
WLST: withdraw life-sustaining therapy
