# Supplementary material for: Intensive End-of-Life Care: Implementation of a Canadian Guideline-Based Order Set for the Withdrawal of Life-Sustaining Therapy in the Intensive Care Unit
Source: Palliat Med Rep. 2025 Apr 10;6(1):161–70. doi: 10.1089/pmr.2024.0091 (PMC12040528; doi:10.1089/pmr.2024.0091)
Supplement: Supplementary Data S1 [file pmr.2024.0091_supp_datas1.docx]

**eSupplement 1. Consolidated Framework for Implementation Research (CIFR) domains and constructs relevant to implementation of Canadian guideline-based order set for the withdrawal of life-sustaining therapy in the ICU.**

| **CFIR Domain** | **CFIR Construct** | **Short Description of CFIR Construct** | **Relevance to our setting** |
| --- | --- | --- | --- |
| 1. Intervention Characteristics | Evidence strength and quality. | Stakeholders’ perceptions of the quality and validity of evidence supporting the belief that the intervention will have desired outcomes. | The ICU healthcare providers value providing best evidence-based care to patients. |
|  | Design quality and packaging | Perceived excellence in how the intervention is bundled, presented, and assembled. | Busy healthcare providers need to have clear and easy to follow materials. |
| 2. Outer Setting | Patient needs and resources | The extent to which patient needs, as well as barriers and facilitators to meet those needs, are accurately known and prioritized by the organization. | The ICU healthcare team values patient and family-centered care. |
| 3. Inner Setting | Implementation Climate | The absorptive capacity for change, shared receptivity of involved individuals to an intervention, and the extent to which use of that intervention will be rewarded, supported, and expected within their organization.   - Tension for change - Compatibility - Relative priority - Learning climate | End of life care in the ICU is emotional and value-laden. It is important to understand the underlying values within the ICU as potential facilitators or barriers to implementation. The study also occurred during the COVID-19 pandemic and healthcare providers may have needed to balance multiple competing priorities. |
|  | Readiness for implementation | Tangible and immediate indicators of organizational commitment to its decision to implement an intervention.   - Available resources - Access to knowledge and information | It is unclear whether sufficient resources are available to support implementation (e.g., nursing time, spiritual care providers and other allied health who provide holistic care)  Introducing new processes to the ICU is generally supported by educational presentations and bedside teaching. |
| 4. Characteristics of Individuals | Knowledge and beliefs about the intervention | Individuals’ attitudes toward and value placed on the intervention as well as familiarity with facts, truths, and principles related to the intervention. | Healthcare providers’ experience with, and values and beliefs about, end of life care may act as a barrier or facilitator to integrating a new intervention for end of life care. |
|  | Individual state of change | Characterization of the phase an individual is in, as he or she progresses toward skilled, enthusiastic, and sustained use of the intervention. |  |
|  | Other personal attributes | A broad construct to include other personal traits such as tolerance of ambiguity, intellectual ability, motivation, values, competence, capacity, and learning style. |  |
| 5. Process | Engaging | Attracting and involving appropriate individuals in the implementation and use of the intervention through a combined strategy of social marketing, education, role modeling, training, and other similar activities. | The Steering Committee, educational initiatives, bedside support, posters, and email communications were utilized as a means of engaging healthcare providers. |
