# Supplementary material for: Intensive End-of-Life Care: Implementation of a Canadian Guideline-Based Order Set for the Withdrawal of Life-Sustaining Therapy in the Intensive Care Unit
Source: Palliat Med Rep. 2025 Apr 10;6(1):161–70. doi: 10.1089/pmr.2024.0091 (PMC12040528; doi:10.1089/pmr.2024.0091)
Supplement: Supplementary Data S2 [file pmr.2024.0091_supp_datas2.docx]

**eSupplement 2. Semi Structured Interview Guides**

**A. Semi Structured Questions for Steering Committee Members**

**[CFIR Framework Domain and Constructs are bolded and enclosed in these brackets]**

Purpose of Semi-Structured Interview: These interviews will occur following the implementation of a physician order set, nursing flowsheet and nursing care plan designed to improve the quality of care during the withdrawal of life-sustaining therapy. The purpose of this interview is to explore factors that may have influenced the implementation process, from the participant’s perspective.

1. Introduction
   1. Introduction of interviewer
   2. Thank participant for their participation: *We appreciate and thank you for taking the time to share your experience and expertise as a member of the ICU End of Life Steering Committee.*
   3. Purpose: *The purpose of this session is to understand the implementation process of the new withdrawal of life-sustaining therapy order set, nursing flowsheet and care plan from your perspective. We are looking to explore the challenges and changes that you see with using these new materials. We will use the results of these interviews to improve this implementation process.*
   4. Use telephone consent and answer any questions they may have.
2. Introduction of the participant: *Could you start by telling me a bit about yourself? What is your profession? How long have you been in practice? Where do you work? Any other training or areas of clinical practice?* **[Characteristics of Individuals – Other Personal Attributes]**
3. Encourage participants to speak about their experience with withdrawal of life-sustaining therapy in the ICU before the implementation of the new order set, flowsheet and care plan: *Can you tell me about your experience seeing patients cared for during the withdrawal of life-sustaining therapy* ***prior*** *to the implementation of the new order set, flowsheet and care plan?*
   1. Probes: *What are some challenges? Areas that are unclear? What are some things your team did well during this time prior to this implementation study?*
4. Encourage participants to speak about their experience with withdrawal of life-sustaining therapy in the ICU after the implementation of the new order set, care plan and flowsheet: *Can you tell me about your experience seeing patients cared for during the withdrawal of life-sustaining therapy* ***after*** *the implementation of the new order set, flowsheet and care plan?*
   1. Probes: *What are some challenges? Areas that are unclear in your practice? What are some things your team does well, or have improved with the new order set, care plan and flowsheet?*
   2. *Do you see a need for the new order set, care plan and flowsheet? Why or why not? Do others see a need for it?* **[Inner Setting - Implementation climate – tension for change]**
5. Implementation process. *Let’s talk about how the order set, flowsheet and care plan were rolled out.*
   1. *How were you involved in the implementation of these documents? ?*
      1. *Probes: Are the learning resources to support implementation sufficient? What else do staff need to support their learning?? Where do staff go if they have questions?*  **[Inner Setting -Readiness for Implementation– Access to Knowledge and Information]**
      2. *How do you, as a supervisor or leader, support the rollout of the new intervention? How do you support learning?* **[Inner Setting – Implementation Climate – Learning Climate]**
      3. *How we make the process of implementing this order set, flowsheet and care plan more engaging?* **[Process - Engaging]**
6. Reaction to new order set and flowsheet. *Now that I understand a bit better how you have been involved in implementing the intervention, let’s talk about what you think about these materials and themselves.*
   1. *What do you think about the rationale or evidence for this order set, flowsheet, and care plan?* **[Intervention characteristics – Evidence strength and quality]**
   2. *Do you feel that the intervention is effective in your ICU? Why or why not?*  **[Characteristics of Individuals - Knowledge and beliefs about intervention]**
   3. *Do [did] you feel your staff are ready to use the new order set, care plan and flowsheet?*  *If not, what do you feel they need to be better prepared?* **[Characteristics of individuals – Individual state of change]**
7. In practice use of the new order set, flowsheet and care plan: *Let’s talk a bit about using the order set, flowsheet and care plan in practice, and what you have seen from the implementation process.*
   1. *What about the printed materials themselves – are they easy to read and use? If not, what should be changed to make them better?* **[Intervention characteristic – design quality and packaging]**
   2. *How well do you think the order set and flowsheet will meet the needs of patients and their families at the end-of-life? In what way?* **[Outer setting – Patient Needs & Resources]**
   3. *How well does the intervention fit with your values and norms, and the values and norms of your colleagues?*  **[Implementation climate – compatibility]**
   4. *How does the intervention affect your staff’s workflow? Do you have enough resources to implement the intervention?* **[Implementation climate – compatibility, Readiness for Implementation - Availability of resources]**
   5. *Has the staff practice changed in caring for patients during the withdrawal of life-sustaining therapy in the ICU? How? Is there a story or personal experience that stands out in your mind that illustrates this?*
8. Feedback. *We just have one last set of questions about what you want to see at the end of the implementation period, and whether you have any other feedback.*
   1. *First, what would you like to know about the effects of the implementation as we are doing this study? How would you like us to get this information to you?* **[Process – Reflecting and Evaluating]**
   2. *Do you have any other suggestions for improvement of the implementation process?*

**B. Semi Structured Questions for Bedside Healthcare Providers (Physicians, RNs, RTs)**

**[CFIR Framework Domain and Constructs are bolded and enclosed in these brackets]**

Purpose of Semi-Structured Interview: These interviews will occur during the implementation of a physician order set, nursing flowsheet and nursing care plan designed to improve the quality of care during the withdrawal of life-sustaining therapy. The purpose of this interview is to explore factors that may influence the implementation process, from the participant’s perspective.

1. Introduction
   1. Introduction of interviewer
   2. Thank participant for their participation: *We appreciate and thank you for taking the time to share your experience and expertise caring for patients during the withdrawal of life-sustaining therapy.*
   3. Purpose: *The purpose of this session is to understand the implementation process of the new withdrawal of life-sustaining therapy order set, nursing flowsheet, and care plan from your perspective as a bedside healthcare professional. We are looking to explore the challenges and changes that you see with using these new materials. We will use the results of these interviews to improve this this implementation process.*
   4. Use telephone consent and answer any questions they may have.
2. Introduction of the participant: *Could you start by telling me a bit about yourself? What is your profession? How long have you been in practice? Where do you work? Any other training or areas of clinical practice?* **[Characteristics of Individuals – Other Personal Attributes]**
3. Encourage participants to speak about their experience with withdrawal of life-sustaining therapy in the ICU before the implementation of the new order set, flowsheet, and care plan: *Can you tell me about your experience caring for patients during the withdrawal of life-sustaining therapy prior to the implementation of the new order set, flowsheet, and care plan?*
   1. Probes: *What are some challenges? Areas that are unclear in your practice? What are some things your team does well during this time?*
4. Encourage participants to speak about their experience with withdrawal of life-sustaining therapy in the ICU after the implementation of the new order set and flowsheet: *Can you tell me about your experience caring for patients during the withdrawal of life-sustaining therapy after the implementation of the new order set, flowsheet, and care plan?*
   1. Probes: *How many patients have you cared for since the new order set started? What are some challenges? Areas that are unclear in your practice? What are some things your team does well, or have improved with the new order set and flowsheet?*
   2. *Do you see a need for the new order set and flowsheet? Why or why not? Do others see a need for it?* **[Inner Setting - Implementation climate – tension for change]**
5. Implementation process. *Let’s talk about how the order set, flowsheet and care plan are being rolled out.*
   1. *How did you learn about the order set, flowsheet, and care plan, and how to use them?*
      1. *Probes: Are these learning resources sufficient? What else would help support your learning? Where do you go if you have questions?*  **[Inner Setting -Readiness for Implementation– Access to Knowledge and Information]**
      2. *What role do your supervisors or leaders in this intervention play in how you learn and integrate the intervention? How do they support you and your learning process?* **[Inner Setting – Implementation Climate – Learning Climate]**
      3. *How we make the process of implementing this order set, flowsheet and care plan more engaging)?* **[Process - Engaging]**
6. Reaction to new order set and flowsheet. *Now that I understand a bit better how you have learned about the order set, flowsheet, and care plan, let’s talk about what you think about these materials and themselves.*
   1. *What do you think about the rationale or evidence for this order set, flowsheet, and care plan?* **[Intervention characteristics – Evidence strength and quality]**
   2. *Do you feel that the intervention will be effective in your ICU? Why or why not?*  **[Characteristics of Individuals - Knowledge and beliefs about intervention]**
   3. *Do [did] you feel ready to use the new order set and flowsheet?*  *If not, what do you feel you need to be better prepared?* **[Characteristics of individuals – Individual state of change]**
7. In practice use of the new order set, flowsheet and care plan: *Let’s talk a bit about using the order set, flowsheet and care plan in practice.*
   1. *What about the printed materials themselves – are they easy to read and use? If not, what should be changed to make them better?* **[Intervention characteristic – design quality and packaging]**
   2. *How well do you think the order set and flowsheet will meet the needs of patients and their families at the end-of-life? In what way?* **[Outer setting – Patient Needs & Resources]**
   3. *How well does the intervention fit with your values and norms, and the values and norms of your colleagues?*  **[Implementation climate – compatibility]**
   4. *How does the intervention affect your workflow? Do you have enough resources to implement the intervention?* **[Implementation climate – compatibility, Readiness for Implementation - Availability of resources]**
   5. *Has your practice changed in caring for patients during the withdrawal of life-sustaining therapy in the ICU? How? Is there a story or personal experience that stands out in your mind that illustrates this?*
8. Feedback. *We just have one last set of questions about what you want to see at the end of the implementation period, and whether you have any other feedback.*
   1. *First, what would you like to know about the effects of the implementation as we are doing this study? How would you like us to get this information to you?* **[Process – Reflecting and Evaluating]**
   2. *Do you have any other suggestions for improvement of the implementation process?*
