# Supplementary material for: Intensive End-of-Life Care: Implementation of a Canadian Guideline-Based Order Set for the Withdrawal of Life-Sustaining Therapy in the Intensive Care Unit
Source: Palliat Med Rep. 2025 Apr 10;6(1):161–70. doi: 10.1089/pmr.2024.0091 (PMC12040528; doi:10.1089/pmr.2024.0091)
Supplement: Supplementary Data S3 [file pmr.2024.0091_supp_datas3.docx]

**eSupplement 3. Feasibility Surveys**

**A. Nursing Feasibility Survey**

**[CFIR Framework Domain and Constructs are bolded and enclosed in these brackets. CFIR Framework Domains and Constructs were removed from the survey distributed to nurses.]**

*This survey will help us evaluate the practical aspects of using the newly developed withdrawal of life-sustaining therapy order set, flowsheet and care plan. Please consider completing this survey after your patient has passed away, or after your shift with a dying patient has ended.*

1. The WLST order set, flowsheet and care plan are supported by good evidence.

**[Intervention Characteristic – Evidence Strength and Quality]**

| 1 – Strongly disagree | 2 – Disagree | 3 – Neutral | 4 – Agree | 5 – Strongly Agree |
| --- | --- | --- | --- | --- |

2. The WLST order set, flowsheet and care plan are easy to read and follow.

**[Intervention Characteristic – Design quality and packaging]**

| 1 – Strongly disagree | 2 – Disagree | 3 – Neutral | 4 – Agree | 5 – Strongly Agree |
| --- | --- | --- | --- | --- |

3a. I have received adequate support to help me implement the WLST order set, flowsheet and care plan.

**[Inner Setting – Implementation Climate – Learning Climate]**

| 1 – Strongly disagree | 2 – Disagree | 3 – Neutral | 4 – Agree | 5 – Strongly Agree |
| --- | --- | --- | --- | --- |

3b. I have been provided with adequate educational materials to help me use the WLST order set, flowsheet and care plan.

**[Inner Setting – Readiness for Implementation – Access to Knowledge and Information]**

| 1 – Strongly disagree | 2 – Disagree | 3 – Neutral | 4 – Agree | 5 – Strongly Agree |
| --- | --- | --- | --- | --- |

4a. The WLST order set, flowsheet and care plan help me to meet the needs of patients at the end of life.

**[Outer Setting – Patient Needs and Resources]**

| 1 – Strongly disagree | 2 – Disagree | 3 – Neutral | 4 – Agree | 5 – Strongly Agree |
| --- | --- | --- | --- | --- |

4b. The WLST order set, flowsheet and care plan help me to meet the needs of my patient’s families when my patient is at the end of life.

**[Outer Setting – Patient Needs and Resources]**

| 1 – Strongly disagree | 2 – Disagree | 3 – Neutral | 4 – Agree | 5 – Strongly Agree |
| --- | --- | --- | --- | --- |

5a. There is a strong need for the WLST order set, flowsheet and care plan.

**[Inner Setting – Implementation Climate – Tension for Change]**

| 1 – Strongly disagree | 2 – Disagree | 3 – Neutral | 4 – Agree | 5 – Strongly Agree |
| --- | --- | --- | --- | --- |

5b. Implementation of the WLST order set, flowsheet and care plan is a priority.

**[Inner Setting – Implementation Climate – Relative priority]**

| 1 – Strongly disagree | 2 – Disagree | 3 – Neutral | 4 – Agree | 5 – Strongly Agree |
| --- | --- | --- | --- | --- |

5c. I feel prepared to use the WLST order set, flowsheet and care plan.

**[Characteristics of Individuals – Individual Stage of Change]**

| 1 – Strongly disagree | 2 – Disagree | 3 – Neutral | 4 – Agree | 5 – Strongly Agree |
| --- | --- | --- | --- | --- |

6. The WLST order set, flowsheet and care plan help me to provide good end-of-life care. **[Characteristics of Individuals – Knowledge and Beliefs about Intervention]**

| 1 – Strongly disagree | 2 – Disagree | 3 – Neutral | 4 – Agree | 5 – Strongly Agree |
| --- | --- | --- | --- | --- |

7a. I felt comfortable following the WLST order set instructions for bolus doses, and titration of narcotics and sedatives.

**[Inner Setting – Implementation Climate – Compatibility]**

| 1 – Strongly disagree | 2 – Disagree | 3 – Neutral | 4 – Agree | 5 – Strongly Agree |
| --- | --- | --- | --- | --- |

7b. I felt comfortable using the WLST care plan as a guide for caring for patients at the end of life and their families.

**[Inner Setting – Implementation Climate – Compatibility]**

| 1 – Strongly disagree | 2 – Disagree | 3 – Neutral | 4 – Agree | 5 – Strongly Agree |
| --- | --- | --- | --- | --- |

8. The WLST order set, flowsheet and care plan fit well with my workflow**.**

**[Inner Setting – Implementation Climate – Compatibility]**

| 1 – Strongly disagree | 2 – Disagree | 3 – Neutral | 4 – Agree | 5 – Strongly Agree |
| --- | --- | --- | --- | --- |

9a. Using the WLST order set, flowsheet and care plan is too intrusive to families.

**[Inner Setting – Implementation Climate – Compatibility]**

| 1 – Strongly disagree | 2 – Disagree | 3 – Neutral | 4 – Agree | 5 – Strongly Agree |
| --- | --- | --- | --- | --- |

9b. There were significant barriers to the implementation of the WLST order set, care plan, and flowsheet (ie. physical environment, work flow, demands on time.)

**[Inner setting – Readiness for Implementation – Available Resources]**

| 1 – Strongly disagree | 2 – Disagree | 3 – Neutral | 4 – Agree | 5 – Strongly Agree |
| --- | --- | --- | --- | --- |

10. I would want to continue using the updated WLST order set, flowsheet and care plan after the 6 month trial implementation process.

**[Inner Setting – Implementation Climate – Compatibility]**

| 1 – No, go back to the old order set and flowsheet. | 2 – Yes, but make some changes (make suggestions in comments section) | 3 – Yes, continue with this current order set and flowsheet. |
| --- | --- | --- |

11. Please rate each of the scales for patient assessment and treatment.

|  | **This scale was easy to assess at the bedside.** | **This scale helped me to titrate medications for pain and sedation.** | **The use of this scale is appropriate in the setting of end-of-life care.** |
| --- | --- | --- | --- |
| **CPOT** | 1 – Strongly disagree  2 – Disagree  3 – Neutral  4 – Agree  5 – Strongly agree | 1 – Strongly disagree  2 – Disagree  3 – Neutral  4 – Agree  5 – Strongly agree | 1 – Strongly disagree  2 – Disagree  3 – Neutral  4 – Agree  5 – Strongly agree |
| **ModRDOS-4** | 1 – Strongly disagree  2 – Disagree  3 – Neutral  4 – Agree  5 – Strongly agree | 1 – Strongly disagree  2 – Disagree  3 – Neutral  4 – Agree  5 – Strongly agree | 1 – Strongly disagree  2 – Disagree  3 – Neutral  4 – Agree  5 – Strongly agree |
| **RASS** | 1 – Strongly disagree  2 – Disagree  3 – Neutral  4 – Agree  5 – Strongly agree | 1 – Strongly disagree  2 – Disagree  3 – Neutral  4 – Agree  5 – Strongly agree | 1 – Strongly disagree  2 – Disagree  3 – Neutral  4 – Agree  5 – Strongly agree |

**Comments:**

______________________________________________________________________________

___________________**___________________________________________________________**

**______________________________________________________________________________**

______________________________________________________________________________

___________________**___________________________________________________________**

**_____________________________________________________________________________**

**B. Respiratory Therapy Feasibility Survey**

**[CFIR Framework Domain and Constructs are bolded and enclosed in these brackets. CFIR Framework Domains and Constructs were removed from the survey distributed to nurses.]**

Please complete this survey based on your experience with the new withdrawal of life-sustaining therapy (WLST) ventilator weaning orders in the newly implemented WLST order set.

RT1. The WLST ventilator weaning orders are supported by good evidence.

**[Intervention Characteristic – Evidence Strength and Quality]**

| 1 – Strongly disagree | 2 – Disagree | 3 – Neutral | 4 – Agree | 5 – Strongly Agree |
| --- | --- | --- | --- | --- |

RT2. The WLST ventilator weaning orders are easy to read and follow.

**[Intervention Characteristic – Design quality and packaging]**

| 1 – Strongly disagree | 2 – Disagree | 3 – Neutral | 4 – Agree | 5 – Strongly Agree |
| --- | --- | --- | --- | --- |

RT3a. I have received adequate support to help me implement the WLST ventilator weaning orders.

**[Inner Setting – Implementation Climate – Learning Climate]**

| 1 – Strongly disagree | 2 – Disagree | 3 – Neutral | 4 – Agree | 5 – Strongly Agree |
| --- | --- | --- | --- | --- |

RT3b. I have been provided with adequate educational materials to help me use the WLST ventilator weaning orders.

**[Inner Setting – Readiness for Implementation – Access to Knowledge and Information]**

| 1 – Strongly disagree | 2 – Disagree | 3 – Neutral | 4 – Agree | 5 – Strongly Agree |
| --- | --- | --- | --- | --- |

RT4a. The WLST ventilator weaning orders help me to meet the needs of patients at the end of life.

**[Outer Setting – Patient Needs and Resources]**

| 1 – Strongly disagree | 2 – Disagree | 3 – Neutral | 4 – Agree | 5 – Strongly Agree |
| --- | --- | --- | --- | --- |

RT4b. The WLST ventilator weaning orders help me to meet the needs of my patient’s families when my patient is at the end of life.

**[Outer Setting – Patient Needs and Resources]**

| 1 – Strongly disagree | 2 – Disagree | 3 – Neutral | 4 – Agree | 5 – Strongly Agree |
| --- | --- | --- | --- | --- |

RT5a. There is a strong need for the WLST ventilator weaning orders.

**[Inner Setting – Implementation Climate – Tension for Change]**

| 1 – Strongly disagree | 2 – Disagree | 3 – Neutral | 4 – Agree | 5 – Strongly Agree |
| --- | --- | --- | --- | --- |

RT5b. Implementation of the WLST ventilator weaning orders is a priority.

**[Inner Setting – Implementation Climate – Relative priority]**

| 1 – Strongly disagree | 2 – Disagree | 3 – Neutral | 4 – Agree | 5 – Strongly Agree |
| --- | --- | --- | --- | --- |

RT5c. I feel prepared to use the WLST ventilator weaning orders.

**[Characteristics of Individuals – Individual Stage of Change]**

| 1 – Strongly disagree | 2 – Disagree | 3 – Neutral | 4 – Agree | 5 – Strongly Agree |
| --- | --- | --- | --- | --- |

RT6. The WLST ventilator weaning orders help me to provide good end-of-life care. **[Characteristics of Individuals – Knowledge and Beliefs about Intervention]**

| 1 – Strongly disagree | 2 – Disagree | 3 – Neutral | 4 – Agree | 5 – Strongly Agree |
| --- | --- | --- | --- | --- |

RT7. I felt comfortable following the WLST ventilator weaning orders**.**

**[Inner Setting – Implementation Climate – Compatibility]**

| 1 – Strongly disagree | 2 – Disagree | 3 – Neutral | 4 – Agree | 5 – Strongly Agree |
| --- | --- | --- | --- | --- |

RT8. The WLST ventilator weaning orders fit well with my workflow.

**[Inner Setting – Implementation Climate – Compatibility]**

| 1 – Strongly disagree | 2 – Disagree | 3 – Neutral | 4 – Agree | 5 – Strongly Agree |
| --- | --- | --- | --- | --- |

RT9a. Using the WLST ventilator weaning orders are too intrusive to families.

**[Inner Setting – Implementation Climate – Compatibility]**

| 1 – Strongly disagree | 2 – Disagree | 3 – Neutral | 4 – Agree | 5 – Strongly Agree |
| --- | --- | --- | --- | --- |

RT9b. There were significant barriers to the implementation of the WLST ventilator weaning orders (ie. physical environment, work flow, demands on time.)

**[Inner setting – Readiness for Implementation – Available Resources]**

| 1 – Strongly disagree | 2 – Disagree | 3 – Neutral | 4 – Agree | 5 – Strongly Agree |
| --- | --- | --- | --- | --- |

RT10. I would want to continue using the updated WLST ventilator weaning orders after the 6 month trial implementation process.

**[Inner Setting – Implementation Climate – Compatibility]**

| 1 – No, go back to the old order set and flow sheet. | 2 – Yes, but make some changes (make suggestions in comments section) | 3 – Yes, continue with this current order set and flow sheet. |
| --- | --- | --- |

RT11. Please tell us about the use of the ModRDOS-4 for ventilator weaning during WLST.

|  | **This scale was easy to assess at the bedside.** | **This scale helped me to titrate medications for pain and sedation.** | **The use of this scale is appropriate in the setting of end-of-life care.** |
| --- | --- | --- | --- |
| **ModRDOS-4** | 1 – Strongly disagree  2 – Disagree  3 – Neutral  4 – Agree  5 – Strongly agree | 1 – Strongly disagree  2 – Disagree  3 – Neutral  4 – Agree  5 – Strongly agree | 1 – Strongly disagree  2 – Disagree  3 – Neutral  4 – Agree  5 – Strongly agree |

**Please provide any comments on how we can improve the WLST ventilator weaning orders or the implementation process.**

______________________________________________________________________________

___________________**___________________________________________________________**

**______________________________________________________________________________**

______________________________________________________________________________
