# Supplementary material for: Intensive End-of-Life Care: Implementation of a Canadian Guideline-Based Order Set for the Withdrawal of Life-Sustaining Therapy in the Intensive Care Unit
Source: Palliat Med Rep. 2025 Apr 10;6(1):161–70. doi: 10.1089/pmr.2024.0091 (PMC12040528; doi:10.1089/pmr.2024.0091)
Supplement: Supplementary Data S4 [file pmr.2024.0091_supp_datas4.pdf]

IMPRINT BELOW THIS LINE

[redacted]

PHYSICIAN'S ORDERS

ALLERGIES:

DATE	TIME	ORDERS AND SIGNATURE	PROCESSED				
		**COMFORT CARE ORDERS: WITHDRAWAL OF LIFE SUPPORT IN ADULT ICU PATIENTS**	TIME	MAR	ICP	REQ	RN

Ensure written order for **Do not call a Code Blue** is present in the health record.

❑ Decrease IV rate to TKO

❑ Discontinue **all** previous medication orders and order analgesia/sedation below. If a neuromuscular blocking agent has been administered to the patient, wait until patient is moving or the nerve stimulator reads 4 twitches out of 4 **before** implementing any changes to ventilatory support. <u>Ensure adequate sedation is available while paralyzed.</u>

❑ Discontinue all previous orders including routine vital signs, pneumatic compression stockings, enteral feeding, TPN, radiographs and laboratory tests. Remove devices not necessary for comfort.

❑ Remove the following if indicated ❑ ECG ❑ Arterial line

- If continuing with ECG monitoring, reduce alarms at the bedside or turn off and use central monitoring when possible

Analgesia: Select one:

❑ Morphine infusion _________mg /hour

For signs of discomfort, give morphine ________mg IV q 5-10min

❑ Fentanyl infusion _________mcg /hour

For signs of discomfort, give fentanyl ________mcg IV q 5-10min

❑ Other:_______________________________________

Sedation: Select one:

❑ Midazolam infusion ________mg / hour

For signs of discomfort, give midazolam _________mg IV q15-20 min

❑ Lorazepam infusion _________ mg / hour

For signs of discomfort, give lorazepam __________mg IV q15-20 min

❑ Other:____

Ventilator:

- Reduce apnea, heater and other ventilator alarms to minimum settings
- Titrate sedation as indicated for discomfort:
  - Reduce $Fi0_2$ to 21% and PEEP to zero
  - Wean IMV to 4 or PS to 5
- When patient is comfortable on IMV rate 4 or PS of 5, select one:

❑ Extubate patient to room air

❑ T-piece with room air (not CPAP mode on ventilator)

Physician signature ________________________ **Date and Time** ____________
