# Supplementary material for: Intensive End-of-Life Care: Implementation of a Canadian Guideline-Based Order Set for the Withdrawal of Life-Sustaining Therapy in the Intensive Care Unit
Source: Palliat Med Rep. 2025 Apr 10;6(1):161–70. doi: 10.1089/pmr.2024.0091 (PMC12040528; doi:10.1089/pmr.2024.0091)
Supplement: Supplementary Data S5 [file pmr.2024.0091_supp_datas5.pdf]

[redacted]

PATIENT IDENTIFICATION

Critical Care Withdrawal of Life Sustaining Therapies (WLST) and Establishment of Palliative/Comfort Care

ACTION: MAR | ICP | REQ | RN

Principle Statement

The goal of this order set is to provide consistent, high quality, symptom-based care at the end of life. This order set is designed for use in critical care settings (ICU, CCU) following a decision to withdraw life-sustaining therapy. Donation after cardiac death (DCD) may follow declaration of death by the medical team, if consented to by the Substitute Decision Maker (SDM) and/or patient.

Prerequisite Care

- Document family meeting between interdisciplinary ICU team and patient (or SDM if patient lacks capacity to make medical decisions). This documentation must confirm consensus on a plan of care that involves WLST and the provision of comfort care.
- Update Resuscitation Care Plan to: Do not call a code – Support natural death
- Consult Donation Coordinator
- Call the Coroner's Office to discuss case, if deemed necessary by circumstances
- **If patient has met organ donation criteria and patient/SDM has consented to donation:**

  ☐ See Organ Donation Management of Adult Organ Donor Order Set (form #102859) for lab collection

Consults

☒ Social Worker
☒ Offer Spiritual or Religious Care if desired by the family
☐ Offer First Nations and Métis Health if desired by the family
☐ Palliative Care (for anticipated difficult symptoms or expected survival greater than 24 hours)
☒ Offer Healing Arts Program [redacted] or Sound of Love Project [redacted]
☐ Ethics consultation

Begin the following steps once the decision is made to move to comfort care in collaboration with the patient and family:

☒ Move patient to private room if possible

☐ Discontinue all monitoring (cardiac monitor, arterial line, blood pressure cuff)

 ☐ For patients proceeding to DCD, continue arterial line monitoring, cardiac monitoring, and continuous SpO_2 monitoring, as directed by Donation Team

☒ Discontinue all previous medications and infusions, except those chosen for comfort (see **Pain and Dyspnea, Anxiety and Agitation**)

 ☒ Continue vasoactive medications at current dose (do not titrate) until family is ready (see **Withdrawal of Physiologic Support**)

☒ Discontinue neuromuscular blockade

- If neuromuscular blocking agents have been used in the past 4 hours, assess train-of-four (TOF). If TOF less than 4/4, consider delaying WLST and alert MD.

☒ Discontinue all investigations including laboratory tests and imaging

☒ Reduce intravenous fluid to TKVO

☒ Discontinue enteral/parenteral feeding, change existing OG/NG to suction to empty stomach, and then clamp.

☒ Remove unnecessary devices from the patient's room

☐ Other: ____________________

PRACTITIONER PRINTED NAME | PRACTITIONER SIGNATURE | DATE/TIME

[redacted]

PATIENT IDENTIFICATION

Critical Care Withdrawal of Life Sustaining Therapies (WLST) and Establishment of Palliative/Comfort Care

ACTION			
MAR	ICP	REQ	RN

Assessment

☒ Critical Care Pain Observation Tool (CPOT) q30minutes until stable then q2H PRN
☒ Modified Respiratory Distress Observation Scale-4 (modRDOS-4) q30minutes until stable then q2H PRN
☒ Richmond Agitation Sedation Scale (RASS) q30minutes until stable then q2H PRN

Pain and Dyspnea

☒ Goal CPOT score 2 or less
☒ Goal modRDOS-4 score 3 or less
☒ Notify MD if unable to attain goal CPOT or modRDOS-4 scores

Select **one** of the following:
☐ dilaudid (**HYDRO**morphone) (**preferred**)

- For infusion, suggested infusion rate of **0.5-2 mg/hour IV** in opioid naïve patients
- For bolus only option, suggested bolus of **0.5-1mg IV** q30min prn in opioid naïve patients

OR

☐ morphine

- For infusion, suggested infusion rate of **1-5mg/hour** IV in opioid naïve patients
- For bolus only option, suggested bolus of **2.5-5mg IV** q30min prn in opioid naïve patients

AND Select **one** of the following:

☐ Bolus ONLY option

- Bolus ____________ to _____________ mg q30minutes prn to achieve goal CPOT and modRDOS-4 score

OR

☐ IV infusion at initial rate of _______________ mg/hour

☒ Give a bolus dose equal to hourly infusion rate q15minutes PRN

- To achieve goal CPOT and modRDOS-4 scores
- Prior to ventilator weaning

☒ Double infusion rate if two PRN doses are given in one hour.

- Infusion range ____________ to __________ mg/hour IV to achieve goal CPOT and modRDOS-4 score

PRACTITIONER PRINTED NAME | PRACTITIONER SIGNATURE | DATE/TIME

[redacted]

PATIENT IDENTIFICATION

Critical Care Withdrawal of Life Sustaining Therapies (WLST) and Establishment of Palliative/Comfort Care

ACTION: MAR | ICP | REQ | RN

Anxiety and Agitation

☒ Optimize **pain and dyspnea** prior to adjusting sedation
☒ Goal RASS Score: ____________
Suggest 0 to -2 if patient is currently able to interact with family and ventilator settings are minimal.
Suggest -4 to -5 if patient is deeply sedated, ventilator settings are high, or high potential for distress.
☒ Notify MD if unable to attain goal RASS score

☐ Continue propofol IV infusion at current rate **and do not titrate** (suggested if patient currently comfortable on propofol, and/or if propofol is being used for seizure control).

For further management of anxiety or agitation,

☒ Start midazolam

Select <u>one</u> of the following:

☐ Bolus ONLY option
- Bolus ___________ to ____________ mg q30minutes prn to achieve goal RASS score

OR

☐ IV infusion at initial rate of ______________ mg/hour (suggested starting dose of **0-2 mg/hour**, or use current dose if patient is already receiving)
  ☒ Give a bolus dose equal to hourly infusion rate q15minutes PRN
  - To achieve goal RASS score

  ☒ Double infusion rate if two PRN doses are given in one hour
  - Infusion range ______________ to ______________ mg/hour IV, to achieve goal RASS score

Respiratory and Oral Care

☒ Provide mouth swabs, ice chips, mouth moisturizer PRN for dry mouth and lips
☒ Suction mouth PRN (avoid deep suctioning)
☒ glycopyrrolate 0.4 mg subcutaneous/IV q2h PRN for respiratory OR oral secretions

PRACTITIONER PRINTED NAME | PRACTITIONER SIGNATURE | DATE/TIME

[redacted]

PATIENT IDENTIFICATION

Critical Care Withdrawal of Life Sustaining Therapies (WLST) and Establishment of Palliative/Comfort Care

ACTION: MAR | ICP | REQ | RN

Withdrawal of Physiologic Support

Initiate when:

- *Family is ready for withdrawal of life support*
- *CPOT, modRDOS~~-4~~ and RASS at goal*

☒ Remove transcutaneous or temporary transvenous pacemaker if in situ.

- If patient has an implanted cardio-defibrillator, consider applying a magnet if it needs to be deactivated.
- Refer to Work Standard: Death or Comfort Measures Only (CMO) for patients with cardiac implanted electronic device (CIED) – Pacemaker, Implantable Cardiac Defibrillator (ICD).

☒ Deactivate mechanical hemodynamic support including intra-aortic balloon pump, ventricular assist device, ECMO

☒ Discontinue all vasoactive medications

Weaning of Ventilation or Respiratory Support

☒ Reduce/disable apnea and other ventilator alarms to minimal settings

☒ Reduce FiO_2 to 0.21 and PEEP to lowest possible value allowed and titrate opioids to modRDOS-4 to less than 4 (suggested time frame 5-30 minutes)

☒ Wean pressure support or pressure control in a step-wise fashion rapidly to 5 cmH_2O and titrate opioids as required to achieve modRDOS-4 goal (suggested time frame 5-30 minutes)

☒ When patient comfortable at minimal ventilator settings

- ☐ Extubate patient (**preferred**)
- ☐ Leave ETT in place
- ☐ Remove non-invasive mask or high flow nasal cannula
- ☒ For patients who are awake, provide O2 PRN via nasal prongs for comfort

Additional Orders

☐ metoclopramide 5-10 mg subcutaneous/IV q6h PRN for nausea or vomiting

☐ acetaminophen 325-650 mg PO/NG/PR q4h PRN for suspected fever

☐ methotrimeprazine ________ to ________ mg subcutaneous/IV q4h PRN for delirium/agitation

☐ haloperidol ________ to ________ mg subcutaneous/IV q _____ h PRN for delirium/agitation

____

____

____

After Death

☒ Notify attending physician

☒ MRP to complete Medical Certificate of Death

☒ Provide family with bereavement pamphlet and support as needed (see Care Plan)

☒ RN to complete Notice of Death release body to funeral home (Refer to policy: Death, Adult and Pediatric care of, release of #1077)

PRACTITIONER PRINTED NAME	PRACTITIONER SIGNATURE	DATE/TIME

[redacted]

Critical Care Withdrawal of Life Sustaining Therapy Record

Page 1 of 2

Date ________________________ Time __________________________

Assessment and Management

Perform CPOT, mod-RDOS4, and RASS scoring q30minutes until stable, then q2h PRN

**Time**										
Heart Rate										
Respiratory Rate										
CPOT (Goal < 2)<br>Titrate opioid to effect										
mod-RDOS4 (Goal < 4)<br>Titrate opioid to effect										
RASS (Target/Current)<br>Titrate sedative to effect once CPOT and modRDOS-4 at goal										
**Medication**										
Opioid (__________)										
Infusion										
PRN										
Sedation (_________)										
Infusion										
PRN										

Modified Respiratory Distress Observation Score4 (modRDOS4)

**Variable**	**0 Points**	**1 Point**	**1.5 Points**	**2 Points**	**2.5 Points**	**4 Points**
Grunting	Absent		Present			
Respiratory rate per minute	≤ 18 breaths			> 18 breaths	> 30 breaths	
Accessory muscle use (clavicle rise)	None			Slight		Pronounced
Paradoxical breathing pattern (abdomen moves in on inspiration)	None	Present				
**Total Score ____ / 9**						

Adapted from Wong et al. Palliative Medicine Reports, 2020 (2.1): 9-14.

**Progress Notes**	
**Time**	**Note**

[redacted]

Critical Care Withdrawal of Life Sustaining Therapy Record

**Progress Notes**	
**Time**	**Note**

[redacted]

Withdrawal of life sustaining therapy care plan

	NURSING DIAGNOSIS	DESIRED OUTCOME	NURSING INTERVENTIONS	Date Initiated<br>ID	Date Discontinued<br>ID
CNS	Risk for pain and discomfort	Patient will experience optimal comfort during end-of-life care and CPOT < 2/8	-CPOT q1h & prn and administer/titrate analgesia to target CPOT < 2/8<br>-Consider additional comfort measures: oral swabs, lip salve, skin care, cool cloths		
CNS	Potential for acute confusion related to end-of-life processes as evidenced by agitation and restlessness	Patient will experience minimal confusion, agitation, and restlessness during end-of-life care	-Assess agitation using RASS q1hr and prn to achieve goal specified by physician<br>-Titrate sedation to target RASS score		
RESP	Ineffective breathing pattern related to end-of-life processes as evidenced by dyspnea, abnormal rate, rhythm, and depth of breathing leading to apnea	Patient will experience minimal dyspnea during end-of-life care and modRDOS-4 of less than or equal to 4	-Assess Modified Respiratory Distress Observation Score-4 (modRDOS-4) q 1hr x 2 when ventilator weaning commenced and prn<br>-Administer oxygen for comfort<br>-Titrate/administer medications to goal of modRDOS-4 <4<br>-Elevate head of bed		
RESP	Ineffective airway clearance related to inability to clear respiratory secretions as evidenced by audible respiratory secretions	Patient will experience minimal audible respiratory secretions during end-of-life care	-Assess for secretions<br>-Clear oral secretions with Yankauer suction prn<br>-Provide oral care prn<br>-Administer medications per order set to minimize respiratory secretion formation		
CARE OF THE FAMILY	FAMILY:<br>Key points for care of the family (from a patient family member)	Families feel supported and empowered.<br><br>Families feel that they have fulfilled their role of advocating for the best care, and protecting their loved one from pain and discomfort	-Remind families that nursing staff are there to support them. Family care is the priority – reinforce that staff are never too busy to answer their questions or concerns.<br>-Reassure families that someone is always watching even when the monitor is off at the bedside<br><br>-Ensure constant communication and education of families. Families may need repeat information in concise, understandable, non-medical language. This includes:<br>-The medication that is given, why it is given, what to expect and how soon<br>-Reassurances that nursing staff will be performing standardized assessments for pain, discomfort, and shortness of breath.<br><br>-Empower families<br>-To monitor effects of medication and let staff know if it isn't effective<br>-To ask about any signs of symptoms their loved one is experiencing<br>-Encourage open visitation<br>-Ensure a private setting<br><br>-After death, ensure Social Work or other support is present, to assist in processing the death.		

PSYCHOSOCIAL	Potential for anxiety and distress related to end of life and fear of the unknown (for both the patient and the family)	Goal: to address the patients psychosocial and spiritual needs	-Support spiritual, religious and cultural beliefs (consider referrals to Spiritual Care, Religious Care, and/or First Nations and Métis Health)<br>-If patient not conscious, speak in a calm, quiet manner, use gentle touch when providing care.<br>-Ask the family what they are worried about at end of life<br>- Ask or offer to facilitate requests for tokens (i.e. Healing Arts Program at SPH, Sound of Love Project at RUH, fingerprints, messages)<br>-Review expected changes in patient status including decreased responsiveness, abnormal breathing patterns including pauses and agonal breathing, heartbeat and pulse changes, and skin appearance as patient nears death.		
	FAMILY:<br>Risk for powerlessness related to end-of-life care as evidenced by anxiety or hesitation around participation in patient care	Family will feel included in patient care and will express comfort in their level of participation during end-of-life care	-Support family member's sense of autonomy by:<br>by giving and accepting information<br>encouraging them to communicate with RN regarding change in patient condition including pain or discomfort<br>supporting them in providing patient care as appropriate		
	FAMILY<br>(Anticipatory) grieving related to end-of-life/impending death	Family will verbalize feelings regarding end-of-life and patient death during end-of-life care	-Collaborate with social worker as appropriate to provide support and a safe space to express grief<br>-Encourage family members to talk with a patient who may be unresponsive<br>-Offer the opportunity to be at the bedside during the dying process and provide support for any decision. Confirm that not being present does not indicate lack of love or caring		
	FAMILY:<br>Readiness for enhanced family coping related to expressed desire to attach meaning to patient's death as evidenced by seeking information regarding organ donation	Family will have sensitive yet timely access to information regarding organ donation during end-of-life care	-Assess family readiness to discuss organ donation if clinically appropriate<br>-Collaborate with physicians, social worker, and donation coordinator to support family in understanding organ donation options as appropriate<br>-Establish rapport with families and empower their decision-making through effective, accurate, and empathetic communication		
PSYCHOSOCIAL	FAMILY:<br>Readiness for enhanced family coping related to expressed desire to attach meaning to patient's death as evidenced by desire for ethnic, cultural, spiritual, or religious practices after death.	Family members will have the opportunity to perform ethnic, cultural, spiritual, or religious practices after patient death.	-Facilitate conversations with family about specific ethnic, cultural, spiritual, or religious rituals that they may wish to arrange or perform after death.<br>-Connect family with social work and/or spiritual care to facilitate above practices<br>-Provide appropriate time and space for above practices<br>-Offer the option to escort the body to the morgue, and/or out of the hospital with funeral personnel (if funeral home known at time of death).		

[redacted]
